# A Colombian strain of *Clostridioides difficile* ribotype 002 induces a highly inflammatory response in a mouse infection model

**DOI:** 10.1080/21505594.2025.2503432

**Published:** 2025-05-12

**Authors:** Juan David Puerta-Arias, Julián Camilo Arango, Carolina Rodríguez-Echeverri, Ariel Arteta, Ángel González

**Affiliations:** a Medical and Experimental Mycology Group, Corporación para Investigaciones Biológicas (CIB-UdeA-UPB-UDES), Medellín, Colombia; b Universidad de Santander (UDES), Facultad de Ciencias Médicas y de la Salud, Bucaramanga, Colombia; c School of Microbiology, Universidad de Antioquia, Medellín, Colombia; d Basic and Applied Microbiology Research Group (MICROBA), School of Microbiology, Universidad de Antioquia, Medellin, Colombia; e Pathology Department, School of Medicine, Universidad de Antioquia, Medellin, Colombia

**Keywords:** *Clostridioides difficile*, CDI, RT002 ribotype, cytokines, inflammatory response

## Abstract

*Clostridioides difficile* causes diarrhea associated with antibiotic use in hospitalized patients. Recent studies have identified that *C. difficile* ribotypes RT002, RT106, and RT591 as the most prevalent circulating strains in Colombia; thus, we aimed to assess the capability of these ribotypes to elicit an inflammatory response during in vivo infection. To achieve this, C57BL/6 mice were treated with cefoperazone (CPZ) for 5 d to develop *C. difficile* infection (CDI) model. Two days post-antibiotic treatment, the mice were orally inoculated with 1 × 10^5^ spores of *C. difficile* strains belonging to ribotypes RT002, RT106, RT591, and RT027 (ATCC strain, used as control). A group of animals was euthanized on day 7 post-infection to determine the bacterial load, total leukocyte number, and chemokines/cytokines levels *in situ*, and for histopathological analysis. RT002-infected groups showed significantly higher bacterial load, CD45+ leukocytes, and RANTES, eotaxin, MCP-1, G-CSF, and IL-2 levels compared to the other groups, suggesting a robust immune response. Furthermore, histopathological analysis of colonic tissue from the group infected with RT002 revealed the presence of an inflammatory response similar to the hypervirulent strain RT027. These results suggest that RT002 of *C. difficile*, one of the main circulating strains in Colombia, can induce a severe inflammatory response, potentially correlating with increased virulence and severity of these strains in CDI cases.

## Introduction

*Clostridioides difficile* (formerly *Clostridium difficile*) infection (CDI) is the most common cause of healthcare-associated infections worldwide; this CDI includes a gamut of clinical manifestations ranging from diarrhea and moderately serious disease to severe pseudomembranous colitis (toxic megacolon) [1,2]. These symptomologies are primarily associated with the production of two toxins, namely TcdA and TcdB, which induce an acute inflammatory response that causes severe damage to the intestinal mucosa. A third toxin, namely CDT or binary toxin, is related to hypervirulent *C. difficile* strains, and has a higher mortality rate [3,4]. Furthermore, prior antibiotic exposure, hospitalization, and advanced age are considered the most important risk factors associated with the development of this condition [1,2].

In recent years, increased mortality and morbidity of CDI have been reported around the world, mainly in North America, Europe, and Asia, attributed to the emergence of hypervirulence and epidemic strains [5,6]. Along these lines, the hypervirulent ribotype RT027/ST1, associated with heightened disease severity and epidemic outbreaks, is the most prevalent ribotype in the United States and some European countries [7–11]. However, different bacterial ribotypes have been reported in several studies worldwide, suggesting a broader epidemiology of CDI that includes a diversity of strains causing infection [12].

It is noteworthy that, in Colombia, the hypervirulent ribotype 027 is rarely reported [13–16]. Moreover, in a study conducted in one of the biggest Colombian cities, a total of 37 ribotypes were identified, with ribotypes RT591/ST141 (20%), RT 106/ST28 (9%) and RT 002/ST8 (7.9%) being the dominant strain types circulating. In addition, four new ribotypes (RT 794, RT 795, RT 804, and 805) and only one 027 ribotype were reported [13,17].

Based on the above observations and to expand current knowledge on the epidemiology and pathogenesis of CDI, we evaluated the inflammatory response elicited by strains belonging to the dominant Colombian *C. difficile* ribotypes (591, 106, and 002) in an in vivo model of infection in comparison with the hypervirulent ribotype 027.

## Methods

### Animals

C57BL/6 eight-week-old male mice were obtained from Sede de Investigación Universitaria (SIU) at Universidad de Antioquia (Medellín, Colombia) and maintained at Corporación para Investigaciones Biológicas (CIB, Medellín, Colombia). The mice were housed in a controlled environment, kept in sterile containers, and provided with acidified water and sterile food at libitum. All animal handling adhered to the national regulations outlined in Colombian law 84/1994, Resolution 8430/1993, and followed the recommendations of the European Union and the Canadian Council on Animal Care.

### Microorganism and growth conditions

This study included *Clostridioides difficile* clinical isolates previously obtained from patients diagnosed with CDI [13,17] (Table 1). These bacterial isolates were grown in the selective medium TCCFA (Taurocholate-Cefoxitin-Cycloserine- Fructose Agar) (Sigma-Aldrich, USA) under anaerobic conditions at 37°C. The *C. difficile* strains belonging to the ribotypes 002, 106, and 591, identified by PCR ribotyping based on capillary electrophoresis, were used for the study [13,17]. *C. difficile* ATCC BAA-1870, a toxigenic and hypervirulent strain associated with higher severity of infection, was obtained from the American Type Culture Collection (ATCC). *C. difficile* spores were prepared as follows: strains were grown overnight in BHIS broth (brain-heart infusion broth supplemented with 0.5% yeast extract and 0.1% cysteine) (Becton Dickinson, NJ, USA). The next day, 100 µl of these overnights were spread onto TY (Tryptone-Yeast extract) (Sigma-Aldrich, USA) agar plates, and plated strains were allowed to grow for 7 d before being removed from the anaerobic chamber. Plates were flooded with 5 ml of cold water or PBS, and bacteria were removed by scraping with a sterile loop. Spores were collected by centrifugation, washed to remove vegetative cell debris, resuspended in PBS, and adjusted to 10^5^ colony-forming units (CFUs) in 100 μL to infect mice by gavage.

**Table 1.** Characteristics of *Clostridioides difficile* strains used in this study.

Strain/Ribotype gene	EIA toxin	Binary toxin	Cytotoxicity in culture (TcdA/TcdB)	PacLoc genes	TcdC gene	Source
027	ND	ND	+/+	ND	+	ATCC
591	Positive	Positive	+/+	+	+	[13,17]
106	Positive	Positive	+/+	+	-	[13,17]
002	Positive	Positive	+/+	+	-	[13,17]

### Clostridioides difficile infection mouse model

Groups of mice (*n* = 6) was treated with the cefoperazone antibiotic (0.5 mg/mL) (MP Biomedicals, USA) administered via drinking water *ad libitum* for 5 d. Following antibiotic treatment, the mice were allowed a 2-d recovery period before infection via oral gavage with the previously described bacterial suspensions. The non-infected control mice received 100 μL of PBS. Six mice were used per experimental group for each ribotype. All animals were observed daily for weight loss, physical aspect (piloerection), spontaneous behavior (inactivity, lack of mobility), mortality, the presence of diarrhea, and other symptoms until sacrificed on day 7 post-infection.

### Colony forming units’ assays

At 7 d post-infection, mice were euthanized by CO_2_ asphyxiation, and cecum content and fecal samples were collected, weighed, and suspended in 1 mL sterile PBS solution. *C. difficile* quantification was performed as previously described [18]. The homogeneous suspensions were diluted (1:10 and 1:100), and 0,1 mL of each dilution was plated on Petri dishes with TCCFA followed by incubation at 37°C for 48 h under anaerobic conditions. The colonies obtained were counted, and the results were expressed as [Log_10_ CFU/g of feces].

### Determination of leukocyte infiltration

Distal colonic tissues were collected, washed with PBS, and divided into two parts with a longitudinal cut. One of the sections obtained from colonic tissue was homogenized in 1 mL sterile 1X PBS with a protease inhibitor cocktail (Roche Applied Science, Germany) using a gentle MACS Dissociator (Miltenyi Biotec, Germany). Homogeneous suspensions were filtered through 100 μm cell strainers (Thermo Fisher Scientific Inc., Waltham, MA, USA) and centrifuged at 1500 rpm, 10°C for 10 min. Aliquots of supernatants were stored at 70°C until being used. The cell pellets were resuspended in RPMI (Gibco, Carlsbad, CA, USA) plus 1% FBS (Sigma-Aldrich, Saint Louis, MO, USA), previously heat-inactivated at 56°C for 30 min, and counted using a hemocytometer. Fc receptors were blocked using a purified rat anti-mouse CD16/CD32 (BD Pharmingen, San Diego, CA, USA). Leukocyte cells CD45+ were determined by flow cytometry using fluorescein isothiocyanate (FITC) anti-CD45 (BD Pharmingen, San Diego, CA, USA), and the respective isotype control. Cells were analyzed using a FACS Canto II system (BD Biosciences, San Jose, CA, USA) and FlowJo V10 software (FlowJo, LLC, Data Analysis Software, Ashland, OR, USA). CD45+ cells were first gated by forward scatter versus side scatter areas (R1), and then CD45+ events were gated from R1. The total number of leukocytes was obtained by multiplying the cell suspension count by the percentage of CD45+ cells.

The other tissue part was submerged in formalin solution [4% formaldehyde solution (EM Science, USA), 0.15 M sodium dihydrogen phosphate (Merck, Germany), 0.11 M sodium hydroxide (Sigma-Aldrich, USA)] and stored until further analysis.

### Cytokine and chemokine measurement

Colonic tissue was homogenized and centrifuged, as previously described. The aliquots of supernatants were used for the determination of cytokines and chemokines by a multiplex assay using a commercial kit and the Luminex 200 system (EMD Millipore, USA). The CC chemokine ligand (CCL)-2 (monocyte chemoattractant protein 1, MCP-1), CCL-3 (macrophage-inflammatory protein 1α, MIP-1α), CCL-4 (MIP-1β), CCL-5 (regulated upon activation normal, RANTES), CCL-11 (Eotaxin), CXC chemokine ligand (CXCL)-1 (keratinocyte chemoattractant, KC), CXCL-2 (MIP-2), CXCL-5 (lipopolysaccharide-induced CXC chemokine, LIX), CXCL-9 (monokine induced by gamma, MIG), leukemia inhibitory factor (LIF), monocyte colony-stimulating factor (M-CSF), granulocyte colony-stimulating factor (G-CSF), IFN-γ, IL-1α, IL-1β, IL-2, IL-6, IL-9, IL-12(p40), IL-12(p70), IL-15, IL-17, tumor necrosis factor-(TNF)-α, and vascular endothelial growth factor (VEGF), were measured.

### Histopathological analysis

As previously described, the colonic tissue fixed with formalin solution was embedded in paraffin, cut, and stained with Hematoxylin and Eosin (H&E) to determine the inflammatory response. The tissue sections were sent to the pathology laboratory of the Universidad Pontificia Bolivariana, in Medellín, Colombia. Thus, a light microscopic evaluation of H&E-stained colonic sections was performed by a pathologist. The pathologist was blinded to the experimental groupings at the time of evaluation, and sections were scored using a previously established system as follows [19]: Edema; 0, no oedema; 1, mild, focal, or multifocal oedema with minimal submucosal expansion; 2, moderate multifocal oedema with moderate submucosal expansion; 3, severe multifocal to coalescing oedema with severe submucosal expansion; 4, same as 3 with diffuse submucosal expansion. Inflammation: 0, no inflammation; 1, minimal, multifocal neutrophilic infiltration; 2, moderate, multifocal neutrophilic infiltration (greater submucosal involvement); 3, severe multifocal to coalescing neutrophilic infiltration (greater submucosal mural involvement); 4, same as 3 with abscesses or extensive transmural involvement. Epithelial damage: 0, no epithelial damage; 1, mild multifocal, superficial damage (vacuolation, increased apoptosis, villus tip attenuation/necrosis); 2, moderate, multifocal superficial damage (same qualitative changes above); 3, severe multifocal to coalescing mucosal damage pseudomembrane formation (intraluminal aggregate of neutrophils and sloughed epithelium in a fibrinous matrix covering eroded or ulcerated mucosa); 4, same as 3 with extensive pseudomembrane or ulcer formation.

### Statistical analysis

The data was analyzed using Graph Pad Prism software version 5 (GraphPad Software, Inc., La Jolla, CA, USA). Medians, and IQR were used to analyze fungal load, flow cytometry, cytokines, and chemokines levels. The Mann–Whitney test was used for comparisons between groups in all developed methodologies. Values of *p* < 0.05 were considered to be significant.

## Results

### A Clostridioides difficile strain belonging to the ribotype RT002 induces a severe CDI-like hypervirulent strain RT027

**Figure 1.**
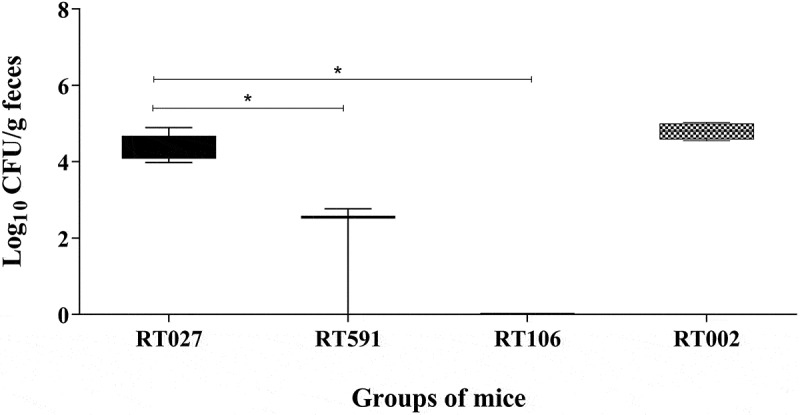
The *Clostridioides difficile* strains belonging to ribotypes 027 and 002 infection are related to an increased bacterial burden in the colon of mice. Bacterial load measurement was performed in the colon tissue of mice infected with different *C. difficile* strains belonging to the ribotypes RT027, RT591, RT106, and RT002 at 7 d post-infection. Data shown represent the median and IQR (*n* = 5–6 mice/group; representative of two independent experiments). A statistically significant increase in bacterial burden was observed in the colon of mice infected with the *C. difficile* strains belonging to the ribotypes RT027 and RT002 (*p < 0.05) compared to the infected mice with the other strains.

Mice were challenged with four different *C. difficile* ribotypes. The bacterial burden in the cecal tissue was quantified at 7 d post-infection. We observed that mice infected with *C. difficile* strains belonging to the ribotypes RT002 and RT027 exhibited a significant and similar high bacterial burden (4.85 and 4.08 Log_10_ CFU/g, respectively), compared to the *C. difficile* strains belonging to the ribotypes RT591 (2.55 Log_10_ CFU/g) and RT106; it is noteworthy that no bacterial colonies were recovered with the latter ribotype (Figure 1). These findings suggest that ribotypes RT002 and RT591 are capable of inducing *C. difficile* infection (CDI) in this experimental model; furthermore, the strain belonging to the ribotype RT002 appears to induce a more severe infection than the hypervirulent strain RT027.

### A Clostridioides difficile strain belonging to the ribotype RT002 induces a high leukocyte infiltrate in the colon

**Figure 2.**
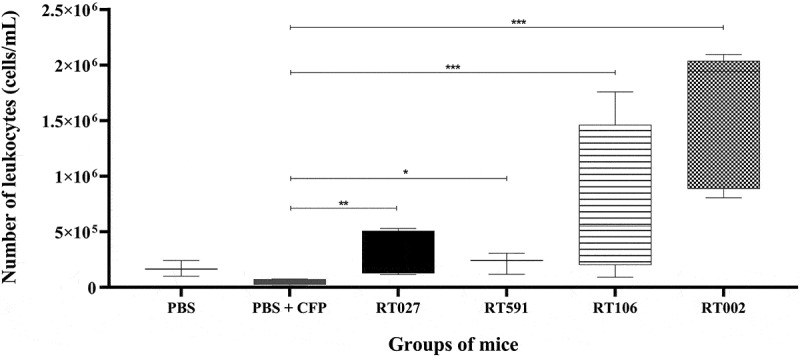
Analysis of the accumulation of leukocytes in the colon of mice infected or uninfected with different *C. difficile* strains belonging to the ribotypes RT027, RT591, RT106, and RT002. The accumulation of leukocytes in the colon was analyzed by using flow cytometry on day 7 post-infection as described in M&M section. Data shown represent the median and IQR (*n* = 5–6 mice/group; representative of two independent experiments). *p < 0.05; **p < 0.01; ***p < 0.001.

To evaluate leukocyte infiltration in a murine model of CDI, the number of CD45+ cells in the distal colonic segment was determined. Comparative analysis of the experimental groups revealed significant differences in the average cell count per microliter. Non-infected (*PBS*) and non-infected, cefoperazone-treated mice (*PBS + CFP*) exhibited lower values, with 0.16 × 10^6^ and 0.05 × 10^6^ cells, respectively. In contrast, mice infected with *C. difficile* strains belonging to the ribotypes RT027, RT591, and RT106 showed a high but modest leukocyte infiltration with a median of 0.3 × 10^6^, 0.24 × 10^6^, and 0.55 × 10^6^ cells, respectively. Notably, mice infected with the *C. difficile* strain belonging to the ribotype RT002 exhibited the highest leukocyte infiltration with a median of 1.94 × 10^6^ cells (Figure 2). These findings suggest that the *C. difficile* strain belonging to the ribotype RT002 induces a marked inflammatory response characterized by a pronounced leukocyte infiltration.

### CDI induced by the C. difficile strain belonging to the ribotype RT002 is associated with higher levels of chemokines and cytokines

**Figure 3.**
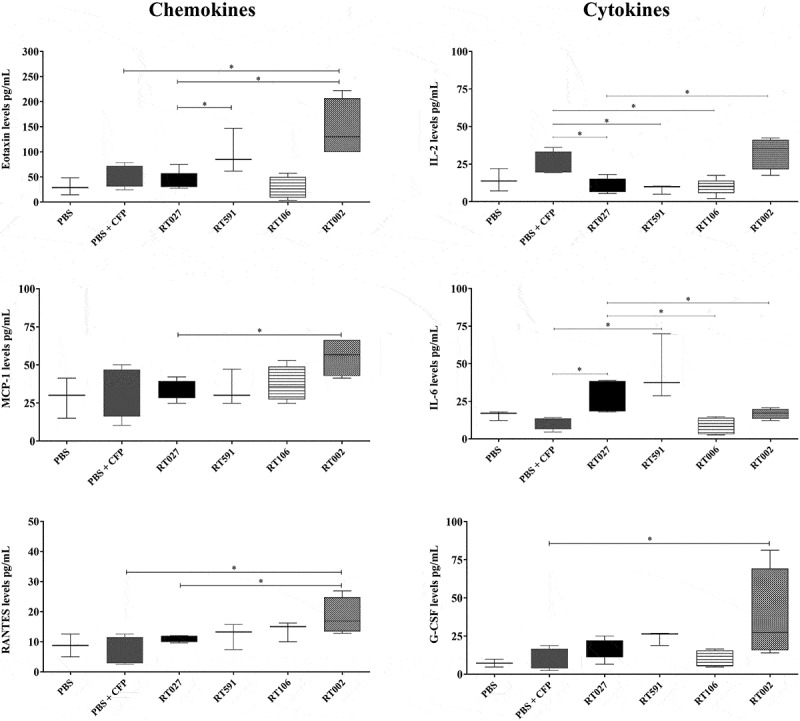
Levels of proinflammatory cytokines and chemokines in the colon of mice infected or uninfected with different *C. difficile* strains belonging to the ribotypes RT027, RT591, RT106, and RT002. Supernatants from colon homogenates of mice sacrificed 7 d after infection were analyzed using a commercial kit and the luminex system, as described in material and methods. Data shown represent the median and IQR (*n* = 5–6 mice/group; representative of two independent experiments). *p <0.05.

Regarding cytokine and chemokine levels, mice infected with the *C. difficile* strain belonging to the ribotype RT002 showed significantly increased levels of eotaxin (CCL11), RANTES (CCL5), MCP-1 (CCL-2), G-CSF, and IL-2 when compared to control (uninfected) mice and with those animals infected with the strains belonging to the ribotypes RT027, RT106, and RT591 (Figure 3). Interestingly, mice infected with the *C. difficile* strain belonging to the RT002 showed significantly lower levels of IL-6 when compared with mice infected with the *C. difficile* strains belonging to the RT027 and RT591 (Figure 3); these findings suggest that the *C. difficile* strain belonging to the ribotype RT002 could be associated with a marked inflammatory response.

### A Clostridioides difficile strain belonging to the ribotype RT002 induces polymorphonuclear infiltration and colonic tissue damage

**Figure 4.**
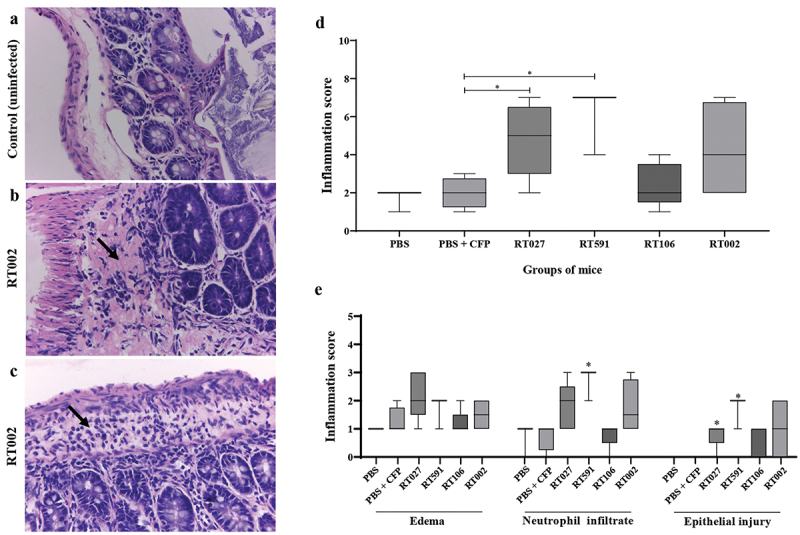
Inflammatory response in colonic tissue of mice infected with the *C. difficile* strain belonging to the ribotype RT002. (a) Representative histology of caecum of healthy uninfected mice (control) with epithelium integrity; (b) colonic tissue of mice infected with a *C. difficile* strain belonging to the ribotype RT002 with edema (arrow) and loss of architecture of epithelium; (c) colonic tissue of mice infected with a *C. difficile* strain belonging to the ribotype RT002 with edema and leukocyte infiltration (arrow); (d) Inflammation histological scores for individual mice in each group. The horizontal lines represent the mean scores for each group of animals. (e) individual evaluation criteria of colonic inflammation. Data shown represent the median and IQR (*n* = 5–6 mice/group; representative of two independent experiments). *p < 0.05 compared to WT-infected mice. Tissue was stained with H&E; magnification 40X. Histopathological micrographs of tissues from animals infected with the other strains belonging to ribotypes RT027, RT591, and RT106 are not shown because after histopathological analyses, the slides and paraffin-embedded tissues were discarded without our consent before documenting the microphotographs.

To confirm the severe inflammatory response induced by the *C. difficile* strain belonging to the ribotype RT002, we examined colonic tissue for histopathological changes in those mice infected with this ribotype. Thus, we observed that the histopathology of the colon of uninfected control mice showed normal tissue with intestinal mucosa of normal thickness and minimal edema (Figure 4(a)); while in mice infected with the *C. difficile* strain belonging to the ribotype RT002 a widened submucosa with edema and infiltration of polymorphonuclear neutrophils was observed (Figure 4(b,c)). Additionally, colonic sections from *C. difficile*-infected and uninfected mice were examined for signs of marked tissue inflammatory response using a score system as described in the Methods section; thus, mice infected with the *C. difficile* strains belonging to the ribotypes RT027, RT591, and RT002 showed a high inflammatory total score in comparison with uninfected mice (control) (Figure 4(d)); similarly, individual analysis of mice infected with the *C. difficile* strains belonging to the ribotypes RT027, RT591, and RT002 showed a high neutrophil infiltration and epithelial damage in comparison with the uninfected mice (control) (Figure 4(e)). Taken together, these data suggest that the *C. difficile* strains belonging to the *ribotypes* RT002, RT027, and RT591 do induce colitis as characterized by an inflammatory response with neutrophil recruitment and tissue damage.

## Discussion

It has been reported that the hypervirulent *C. difficile* RT027 is associated with epidemic CDI outbreaks globally, mainly in the USA and some European countries. Nonetheless, this strain has only been sporadically reported in Colombia. Instead, ribotypes RT591, RT106, and RT002 are the most prevalent ribotypes circulating in the region [13,17]. Herein, the virulence capability of the *C. difficile* strains belonging to the ribotypes RT591, RT106, and RT002 was further investigated in a model *in vivo* and compared with the hypervirulent ribotype RT027. We observed that among the strains evaluated, the *C. difficile* strain belonging to the ribotype RT002 exhibited the highest bacterial burden accompanied by a significant leukocyte infiltration at the colonic tissue and increased levels of chemokines and cytokines, including eotaxin (CCL11), RANTES (CCL5), MCP-1 (CCL2), G-CSF, and IL-2, in comparison to the hypervirulent RT027 and the other strains evaluated.

Notably, the *C. difficile* ribotype 002 has been emerging as the most common ribotype causing CDI in Hong Kong [20–22], Scotland [23], Hungary [24], Kuwait [25], Australia [26–28], New Zealand [29], and Europe [30], with reports associating it with increased mortality [12]. Recently, Dauby et al. reported a fatal case of community-acquired *C. difficile* ribotype 002 bacteremia [31]; these findings could suggest that this ribotype exhibits a high degree of virulence. In another study, Dost et al. sequenced 298 C. *difficile* RT002/ST8 strains representing a new European genome collection, with strains from Germany, Denmark, France, and Portugal; moreover, they complemented their findings with genomes from the public Enterobase database and found a close genetic relatedness among the studied ST8 genomes and a diverse array of antimicrobial resistance genes. Additionally, clonal isolates across different One Health sectors (humans, animals, environment, and food) were found, indicating the importance of this ribotype in the epidemiology of CDI as well as in the context of One Health [30].

Among the virulence mechanisms exhibited by *C. difficile* associated with the clinical outcomes of CDI, higher levels of toxins and sporulation rates have been related with the hypervirulent ribotype RT027 [32,33]. Thus, Kong et al. observed a significantly higher toxin A and toxin B production in ribotype 002 than other common ribotypes circulating in Hong Kong [12]. The *C. difficile* strain belonging to the RT002 analyzed in the present study displayed toxin production, and cytotoxicity and contained the PacLoc *tcdA*+ and *tcdB*+ genes [17].

Furthermore, it has been suggested that the severity of CDI as well as the greater spread or persistence in hospitals of the RT027 are associated with the rate and enhanced sporulation capacity of this strain [34–36]. On the same token, it has also been reported that the higher prevalence and higher mortality reported in Hong Kong attributed to the RT002 may be associated with its sporulation capacity and environmental persistence [12,20]. Although in the present study, we did not evaluate the sporulation capacity of the circulating RT002, this biological mechanism could not be ruled out.

Additionally, we observed that the *C. difficile* strains belonging to the ribotype RT002 were able to cause important tissue damage with substantial neutrophilic infiltration and mucosal alteration, consistent with previous studies [12].

These findings indicate that the strain belonging to the *C. difficile* RT002, one of the most common ribotypes circulating in Colombia, exhibits a higher virulent capability and must be considered an important emergent *C. difficile* ribotype causing a severe CDI. Thus, this virulence characteristic was associated with higher bacterial load, pronounced inflammatory response and tissue damage *in situ*, like the hypervirulent ribotype 027.

It is important to note that one of the main limitations of this study was the use of a single representative strain of each of the three main ribotypes circulating in Colombia, so the results of this study cannot be generalized and extrapolated, and it is therefore necessary to conduct more studies that allow the analysis of a sizeable number of strains. Moreover, we did not determine other parameters associated with CDI such as weight loss or other clinical signs associated with severe infection, and since the animals did not succumb, we only limited to immunological and histological parameters.

Additional studies exploring other virulence factors, including the sporulation capacity, and the association with the CDI severity and mortality caused by this RT002, are needed. Furthermore, healthcare providers and infection control teams should be alerted to mitigate potential outbreaks caused by this *C. difficile* ribotype (RT002).

## Supplementary Material

ARRIVE _Checklist .pdf

## Data Availability

The data supporting the findings of this study are available at Figshare (https://doi.org/10.6084/m9.figshare.28501952.v1).
